# Progress in Medicine

**Published:** 1898-02-19

**Authors:** 


### PROGRESS IN MEDICINE.

### RENAL MEDICINE.

(*Continued from page* 329.)

Paulinoff,[1] in discussing the classification of the various forms of nephritis, argues that there are two chronic types due to scarlatina—a parenchymatous when the duration of the disease has been short, and an interstitial when it has lasted, say, from twelve to eighteen years. Thus the origin is often forgotten, and many cases set down as idiopathic are really due to a long-forgotten attack of scarlatina. Senator also took part in the Moscow debate, and laid down that chronic nephritis is due either to the effects of acute nephritis or to latent, long-continued inflammations. The disease, indeed, may continue to progress long after the original cause has ceased to act, while the causes producing an acute disease may attack either the connective tissue or the parenchyma. There is also a primary renal sclerosis which affects first the connective tissue, and finally a type which arises through an inadequate blood supply to the kidney, either in old age or in severe anæmia. Somewhat similar are the effects of arterio-sclerosis, which may cause nephritis as well as follow it. Brault goes still further, and abolishes all distinctions between the varieties of nephritis except those due to the duration of the disease and the original infection, or rather to the special microbic or mineral toxin which originated it. Thus he recognises transient, acute, sub-acute, and chronic forms, while the pathological appearances appear to him largely accidental.

Investigations are still being made on the methylene blue test for the permeability of the kidney, a test which seems of some value both in medicine and in surgery, since it shows whether the kidneys are acting normally and excreting noxious matters. Achard and Castaigne[2] have applied it to 50 fresh cases, but it is clear that demonstrable lesions are not found in all cases where excretion is delayed. If a circumscribed lesion is present permeability may remain perfect, provided the rest of the organ remains healthy. In some cases a colourless product of the methylene blue is passed with or before the excretion of the pigment. This chromogen when heated in the urine with acetic acid gives a green colour. As to its importance, the authors consider that if both the blue and the chromogen are delayed there is defective permeability. If the blue only is delayed the lesion is less serious, and may, indeed, be functional and temporary. It may be of use here to give the method of applying the test which was noticed in a previous paper. A deep injection is made into the muscles or connective tissue of ·05 grams, or 1 c.c. of a one-in-twenty solution of methylene blue, when it is desired to test the condition of the kidneys. The bladder should be emptied just before the injection, and again at half an hour, one, two, and three hours afterwards. In a healthy man the water is coloured with the blue in half an hour. The tint reaches its maximum in three or four hours, and vanishes after 50 hours. In kidney disease, with the exception of acute epithelial nephritis, a constant symptom is the delayed appearance of the blue in the urine.

The chief supporters of the theory of functional albuminurias without kidney lesions seem to be found in France. Robin[3] defines a dyspeptic variety in patients with gastric hyperæsthenia, large appetites, and constipation. They are emaciated and suffer from vertigo, but never show any œdema. The albumen is never found before breakfast, consists exclusively of serin, and is unaccompanied by casts. If treated in the early stage while it is intermittent it is invariably cured, but when it has become permanent only about a third of the cases recover. Milk diet may be necessary for a time, and afterwards the amount of meat or of vegetable food must be reduced, as the albumen is found to be decreased, by a diet modified in one direction or the other. He believes that the condition is due not to toxins, but to imperfectly metabolised albumen absorbed from the food, which acts on the kidney as a foreign body. D. D. Stewart[4] gives an account of certain cases of chronic nephritis without albumen. These are not merely early stages of granular kidney, or cases of arterio-sclerosis where the kidney affection, if any, is secondary, but show a scanty unconcentrated urine and symptoms of uræmia, a diminution of urea, and the constant presence of casts. There may be constant headaches and attacks of vertigo, and lumbar pain. An examination of the kidney in a person who suffered in this manner showed a sclerosis in the vessels near the glomeruli which extended to the tubules. Free uric acid crystals were seen in the urine shortly after it was voided, and the suggestion is made that, through some such renal inadequacy as is seen in these cases, the first stage in the progress of gout is brought about, though the main purpose of the paper is to emphasise the existence of these types of kidney lesions without albuminuria.

Further investigations on the production of uræmia have been made by Herter,[5] showing that the blood after ligature of the renal vessels becomes increasingly toxic. The toxicity of the serum was destroyed by heating to a temperature between 60 deg. and 70 deg. C. It could not be due to potassium salts, of which the serum contains little; nor to urea and extractives, for these require a much higher temperature for their destruction; nor was the poison stored up in the liver, but rather in the skeletal muscles. The intestines of the animals thus examined showed marked patches of congestion, and a similar appearance could be produced by injections of the serum from uræmic patients, or of quantities of urea. Yet little urea was found in the intestines of the animals with ligatured vessels, though the quantity in the blood rose from two to ten times the normal. As a practical inference, the writer was led to believe that bleeding would be found of great value in uræmic dyspnœa, especially when there was good heart action, high tension, and only moderate anæmia. The danger of driving out of the muscles into the blood quantities of the uræmic poisons in gouty and other patients is another corollary, which is insisted on in the *Lancet*.[5] Massage applied to patients with damaged kidneys without due consideration is very apt to injure the kidneys still further, and even to set up uræmia, though it may relieve a certain amount of pain and stiffness in the limbs. Of late such cases have been so numerous that "massage kidney" bids fair to be a recognised disease. Vitrac[6] recently described a case which may explain some few instances of hæmaturia without apparent cause. The kidneys and bladder seemed quite healthy, but a small cancerous nodule was found in the rectum, and when this was excised the hæmorrhage ceased. With its recurrence the bleeding returned, but when the rectum was extirpated it again disappeared. The connection of the vesical vessels with those of the rectum probably led in this case to a vesical hæmorrhage, though in the absence of bladder symptoms it might have been referred to some congestion of the kidney. Another rare cause of hæmaturia was noted by Edebohls[7] in a traveller from South Africa. This was the Hæmatobium Bilharzii. The urine contained many blood corpuscles, pus cells, casts, and the ova of the parasite, which were seen to hatch when placed in water. Diminution of urea was regarded by Rommelaere as an important sign of malignancy in tumours, but this has been pretty thoroughly disproved by Reynes and others, who show that it has no diagnostic value in those affections. Reynes[8] finds it especially in a class of persons of infantile appearance, pale complexion, cold extremities, poor appetite and digestion, and inability to bear fatigue. In this he agrees with the description of renal inadequacy given by Sir Andrew Clark, which, however, has not found much favour in this country. He notices that there is great improvement when the patients are treated by rest, strengthening diet, and tepid salt douches, which increase enormously the excretion of urea and of urine. As a standard of disease, he regards all persons who pass less than 12 grams of urea as suffering from hypoazoturia, and he directs attention to the opinions of many surgeons that serious operations are peculiarly fatal in such patients.

[1] Med. Week., Sept. 17. [2] N.Y. Med. Jour., Oct. 16. [3] Med. Week, Aug. 20. [4] Lancet, Sept. 4. [5] Med. Record, Aug. 21. [6] Lancet, Oct. 2. [7] N.Y. Med. Jour., Ap. 3. [8] Med. Week, July 30.

### NEUROLOGY.

(*Continued from page* 345.)

**Epilepsy.**—Andriezen[1] has recently made most important researches on the pathology of epileptic idiocy and imbecility in the child. He finds two lesions—(*a*) A diffuse sclerosis or overgrowth of the neuroglia fibre cells in the brain substance; (*b*) a coextensive change in the nerve cells—of two kinds, positive and negative. The negative changes consist in a defective development (fewness and slenderness) of protoplasmic processes, whilst the positive changes consist of an increase in the amount and diffusion of pigment throughout the cell body, especially its basal part. Later changes were a gradual destruction and atrophy of the cell-processes, consequent upon or coextensive with the further overgrowth of the glia cells. This double process may affect predominantly this or that area of the brain, frequently in territories corresponding to a particular vascular distribution.

In cases of epilepsy supervening in adult life, after the brain cells have attained complete development, the changes found in the nerve cells were only of the positive kind above, with frequently, in addition, intranuclear vacuolation of the cortical cells.

The significance of these changes is the more striking on comparing them with those found in the brains of simple, non-epileptic idiots and imbeciles; for in the latter class of cases the brain convolutions are plump and well formed, though simple in arrangement, and on microscopic examination it is seen that the changes in the nerve cells are only of a negative kind, *i.e.*, the cells are plump and embryonic-looking, with few protoplasmic processes, whilst there is no evidence of overgrowth of the neuroglia, or of destruction of the processes of the nerve cells.

Pierce Clark[2] defines epilepsy as a disease characterised by a periodic disordered state of consciousness, which may or may not be preceded, attended, or followed by a perceptible or imperceptible muscular contraction. The disorder of consciousness may be one of three kinds, the commonest of which is entire loss. Or it may be a partial loss only, especially when large doses of sedatives are being taken and the cortical discharge is but slight. Or, thirdly, the disorder of consciousness may be one of exaltation, as seen in rare cases of somnambulistic epilepsy. The mental failure so often seen in epileptics is easily explained on the basis that these rapid disordered states of consciousness are but partially recovered from after each attack, and the memory is the first to suffer irreparably. Progressive mental failure is the typical mental change of epilepsy. True melancholia is rare, as are also constant systematised delusions.

Bromides are the sheet-anchor of nearly every physician in cases of epilepsy. Occasionally they are entirely ineffectual, and in other cases, though the fits are stopped, symptoms of bromism may be such as to make one hesitate to continue them. But the fear of status epilepticus may prevent this. However, Petersen[3] shows that gradual or even sudden reduction of the quantity of bromides in long saturated cases does not increase the liability of status epilepticus occurring, and does ameliorate the patient's condition in almost every instance, in that the number of fits is lessened, their severity is diminished, and the general health and mental condition are improved.

The successful treatment of the status epilepticus is one of the most difficult and trying possible. Pierce Clark,[4] of the Craig Epileptic Colony, has never found any benefit from the administration of nitrite of amyl. He says that chloroform can be used with great advantage at the beginning of the convulsive stage; it should not be used in the sleep or stupor stage. He finds that though chloral by itself does little good, yet a combination of chloral, morphia, and bromide, if given early, is most efficacious—in almost all cases no more seizures occur, or at most but two or three.

**Locomotor Ataxy.**—Some years ago the subject of locomotor ataxy was thought to be exhausted, and its symptomatology and pathology quite clear. But from time to time additions to our knowledge are made which alter our ideas in some degree, and show new and unexpected features. Thus the investigations of Laehrs,[5] which have been continued by Patrick,[6] and more recently by Bonar,[7] into the distribution of the skin anæsthesia, show that its distribution does not correspond with that of the peripheral nerves. Thus trunk anæsthesia is very common, occurring in from 80 to 90 per cent. of all cases. It is not very marked, and relates particularly to the perception of touch rather than pain. Its location is at about the level of the nipple, and takes the form of a band reaching from the spine to the middle line in front; it is nearly always bilateral, though the bands may be at different levels and of unequal widths. These bands have a more horizontal position than the ribs. If it extends upwards, as soon as the third intercostal space is reached it begins to extend on to the arm, at first affecting only the inner surface below the axilla, and then gradually covering the whole inner border before spreading towards the radial side. This distribution corresponds to the gradual upward invasion of spinal segments, and a similar progress downwards is sometimes to be traced. This segmental distribution of the anæsthetic areas in tabes is a discovery of a good deal of importance in regard to our theories of the nature of the posterior column sclerosis in this disease; evidently its seat, as regards individual fibres, depends on the segments they innervate, and not on the particular posterior root by which they have entered the cord. It is not merely a question of ascending degeneration up the cord from affection of individual posterior roots or posterior root ganglia.

As the border of the anæsthetic zone is often hyperæsthetic, and in this border the cutaneous reflexes are particularly lively, it is thus easily possible to account for differences of opinion regarding the activity or absence of superficial reflexes in tabes. If the skin area, say, of the cremaster reflex is just at the border of an anæsthetic zone it will be exaggerated, if within it will be lost.

The discovery of such areas of anæsthesia may be of practical importance. Thus a man with a history of syphilis had both knee-jerks gone, and slight swaying of the body when the eyes were closed. There were no other symptoms. The discovery of an area of tactile anæsthesia on the chest clinched the diagnosis of tabes.

**Lumbar Puncture.**—Fresh evidence of the value of lumbar puncture in diagnosis has recently been brought forward by Leuhartz,[8] Fürbringer,[9] and Wilms.[10] It appears to be useful more particularly in the diagnosis of tuberculous from idiopathic cerebro-spinal meningitis. Thus the tubercle bacillus was found in 21 out of 46 cases of tuberculous meningitis, and out of 24 cases of the non-tuberculous variety Weichselbaum's diplococcus was found in 13, and Fränkel's diplococcus in 9 cases. It is evident, then, that lumbar tapping does not in all cases allow of a differential diagnosis being made between tuberculous and non-tuberculous cases. This is confirmed by the fact that Weichelselbaum's diplococcus was found in one case of tuberculous meningitis (as proved by an autopsy). A positive result—the finding of tubercle bacilli—settles the diagnosis, but a negative result does not exclude it.

Lumbar tapping does not exert any unfavourable clinical influence; on the contrary, it often relieves the headache. Lumbar tapping may also be of use in the diagnosis of intracranial or spinal effusions of blood, by traumatism, or hæmatoma of the dura mater. The blood withdrawn may be pure or nearly so, or consist merely of liquid with a red stain—*e.g.*, intracranial hæmorrhage of traumatic origin was thus diagnosed in a case of delirium tremens, when otherwise it would have escaped notice.

**Erythromelalgia.**—Prentiss[11] has published an account of two cases of the rare disease red neuralgia or erythromelalgia, which was first described by Weir Mitchell. The disease—fortunately a rare one, for treatment is unavailing—is characterised by redness, intense burning pain, and, at the beginning, rise of temperature, with later fall in temperature. It is the feet which are affected as a rule, sometimes the hands as well, and rarely the hands only. The pain usually commences in the ball of the foot, or the heel, or the great toe, and extends to the sole and dorsum; in bad cases it is more or less at all times. One great peculiarity is that it is worse in summer and in heat, and is eased by cold. There is little or no difference in colour until the foot hangs down, when it becomes of a rose-red and hot, and the arteries throb. No gangrene ever takes place, and the disease is asymmetrical. It is most likely to be mistaken for Raynaud's disease, but on comparison it is evident that most marked differences exist, for in the latter the affected parts are either bloodless and white or of a deep dusky colour, with a lowered temperature. Gangrene may take place, and the affection is generally symmetrical. The pathology of erythromelalgia is quite unknown; it may be either due to central disease, or to a peripheral neuritis affecting principally or only the smaller nerves or nerve endings.

The treatment is chiefly that of the general health and of the pain: for the latter purpose phenacetin protected by caffein seems of first value, but hypodermic injections of morphia may become imperative for the most violent paroxysms. One case has been reported in which cure resulted apparently from the administration of arsenic. If these means fail, and from the cases recorded it is probable that this will be so, surgical treatment may be adopted. One case has been reported in which cure resulted from stretching and exsection of the nerves running to the foot. In another case, however, death from gangrene resulted from a similar procedure.

**Ascending Degeneration in Mixed Nerves.**—It has been known for some years that if a limb be amputated the nerves of the stump become in course of time much reduced in size. But exactly what happens is a matter of dispute. Some authorities believe that the reduction in size is due to simple atrophy of the nerve-fibres; others think that whilst the myelin of the nerve-fibres suffers the axis cylinder remains intact. A further question is whether this process is a degeneration or an inflammation.

Flemming[12] has recently published a valuable account of experiments undertaken in the hope of settling these questions. A nerve trunk—generally the sciatic—was severed, and the resulting changes in the central end investigated at various intervals subsequently. It was found that the slightest sepsis of a wound, if a nerve lie exposed in that wound and the nerve-fibres have been divided, leads to an ascending degeneration which may spread up to the spinal cord. This ascending degeneration is due to inflammation, as is shown by the presence of exudation with leucocytes in addition to degenerated fibres and segmentation of myelin.

On the other hand, if perfect sepsis were attained, it was found that: (1) The smaller nerve-fibres of the nerve appear to suffer first and most—they degenerate a certain distance upwards, and connective tissue replaces the severed fibres; (2) the larger nerve-fibres, though they do suffer to a certain extent, only show a simple atrophic change, and the myelin suffers more objectively than the axis cylinders; and (3) there is a very gradual thickening of connective tissue, perineurium and endoneurium, which takes the place of atrophied fibres. Flemming is inclined to think, then, that ascending degenerative changes in a severed nerve, other than the slow atrophic processes just described, are of septic origin.

**Crossed Adductor Jerk.**—Stewart[13] has recently reported the results of a careful examination of a case in which, when the right patellar tendon jerk was tapped, two jerks occurred, one of the right quadriceps extensor, the other of the left adductor muscles, and conversely when the left patellar tendon was tapped. These movements were recorded on a moving plate, and the tracings show clearly that the crossed adductor jerk occurs at an interval distinctly later than the knee-jerk. The author concludes that the crossed jerk is a true reflex, and, unlike the knee-jerk, not the result of a direct response of the adductor muscles.

[1] Andriezen, Brit. Med. Jour., May 1, 1897. [2] Pierce Clark, Med. Record, Sep. 11, 1897. [3] Petersen, Med. Record, Sep. 25, 1897. [4] Pierce Clark, Pediatrics, August, 1897. [5] Laehrs, Archiv. für Psych., XXVII. [6] Patrick, New York Med. Jour., Feb. 6, 1897. [7] Bonar, New York Med. Record, May 22, 1897. [8] Lenhartz, Med. Week, July 2, 1897. [9] Fürbinger, Med. Week, July 2, 1897. [10] Wilms, Münch. Med. Woch, Jan. 19, 1897. [11] Prentiss, New York Med. Record, July 10, 1897. [12] Flemming, Edin. Med. Jour., Jan., 1897. [13] Stewart, Jour. of Phys., 1897.

